# A phase II trial of BAY 43-9006 (sorafenib) (NSC-724772) in patients with relapsing and resistant multiple myeloma: SWOG S0434

**DOI:** 10.1002/cam4.276

**Published:** 2014-06-10

**Authors:** Gordan Srkalovic, Mohamad A Hussein, Antje Hoering, Jeffrey A Zonder, Leslie L Popplewell, Harsha Trivedi, Sandy Mazzoni, Rachel Sexton, Robert Z Orlowski, Bart Barlogie

**Affiliations:** 1 Sparrow Cancer CenterLansing, Michigan; 2 Celgene CorporationSummit, New Jersey; 3 Southwest Oncology Group Statistical CenterSeattle, Washington; 4 Karmanos Cancer InstituteDetroit, Michigan; 5 City of Hope Medical CenterDuarte, California; 6 Michigan State University College of Osteopathic MedicineEast Lansing, Michigan; 7 The University of Texas MD Anderson Cancer CenterHouston, Texas; 8 University of Arkansas for Medical SciencesLittle Rock, Arkansas

**Keywords:** Multiple myeloma, Raf oncogene, Ras oncogene, sorafenib, VEGF

## Abstract

The authors assessed the overall response rate, including confirmed complete response (CR) and partial response, in patients with relapsed/refractory multiple myeloma treated with sorafenib. Qualitative and quantitative toxicities associated with this regimen were evaluated. Patients were eligible if they had a confirmed diagnosis of refractory or relapsed (RR) multiple myeloma (MM) with measurable monoclonal protein. Patients had to have adequate renal, hepatic, hematologic, and cardiac function with a Zubrod performance status of 0–2. Patients were given 400 mg sorafenib by mouth twice daily for 28-day treatment cycles. These patients were followed up for a maximum of 3 years to assess responses and adverse events. Twenty-three patients were enrolled. Of these, five were found to be ineligible for the following reasons: four had insufficient documentation of the baseline disease and one patient did not have measurable disease. All eighteen eligible patients were evaluable for toxicities. Three patients experienced grade 4 toxicities: one with thrombocytopenia, one with anemia, and one with renal failure. Four of the eighteen eligible patients were not assessable for response due to removal from protocol treatment prior to adequate disease assessment. Specifically, three were removed for either grade 4 toxicity or progression of disease and one was removed per patient choice (due to reasons unrelated to treatment). Of the 18 patients who were assessed for toxicities, 5 (27.8%) received at least one fully dosed cycle, 2 (11.1%) of whom had all cycles fully dosed. No responses were observed on this study of the 14 patients who were assessable for response. All patients have discontinued protocol treatment as of August 2008. Overall survival at 12 months was 50% (95% CI 27–73%) and median progression-free survival was 1.2 months (95% CI 1.0–5.4). The trial did not exhibit activity by the International Uniform Response Criteria for MM. Further research should focus on combination therapy of sorafenib with standard treatments in selected patients with RR MM.

## Introduction

Multiple myeloma (MM) is a neoplastic plasma cell disorder characterized by clonal proliferation of malignant plasma cells in the bone marrow microenvironment. It is an age-dependent cancer where, characteristically, the affected plasma cells retain their potential for proliferation 1. It accounts for ∼1% of neoplastic diseases and 13% of hematologic malignancies 2. In the year 2012, almost 22,000 new cases were reported, making this disease the second highest diagnosed blood neoplasm 3. Although advancements have been made in the treatment (average life expectancy has risen from 3 to 6 years) this still remains an incurable disease, with nearly all patients relapsing after initial treatment 4.

Resistance is in part due to this disease's relationship with its microenvironment 5. MM is involved in derailing a complex cascade of signaling pathways and compromising different subsets of cells leading to this cancer's adaptability 6.

The proliferation of MM tumors is dependent on changes to the immediate microenvironment, which is heavily influenced by tumor cell interaction with nearby marrow cells. Several pathways are upregulated in myeloma cells, such as Ras/Raf, along with elevated vascular endothelial growth factor (VEGF) allowing the tumor to expand more rapidly 5. The inhibition of VEGF is important in the reduction of tumors, which rely on increased vascularization for sustained growth 7. It is these specific interactions that are now being researched and targeted in hopes of developing better and more effective drug treatments.

A high incidence of Ras mutations exists in plasma cell disorders which may be upward of 35–50% 8. This increases as the stage of the disease advances and becomes more resistant. Recently, whole genome sequencing of myeloma samples also revealed mutations in the BRAF kinase itself 9. Inhibition of the Raf kinase, as well as the angiogenic *β*-signaling pathway, could provide a useful new approach for the treatment of MM. Moreover, preclinical studies have validated sorafenib as an agent with antimyeloma activity in both in vitro and in vivo model systems 5,10. We present here the data from the study conducted by Southwest Oncology Group (SWOG) evaluating the effect of sorafenib as a single agent in relapsed refractory MM patients (S0434).

## Patients and Methods

### Patient selection

Eligibility requirements included men and women with a confirmed diagnosis of previously treated, active MM. Active MM was defined based on the Durie–Salmon major and minor criteria 11.

Patients in the trial must have had relapsed or resistant disease, defined as relapsing after autologous stem cell transplantation or either relapsing or resistant after greater than one line of prior myeloma therapy. A minimum of 42 days must have passed since prior transplantation. Patients were required to be off myelosuppressive chemotherapy for at least 21 days (at least 6 weeks for nitrosoureas) and greater than 14 days for nonmyelosuppressive chemotherapy and radiation prior to registration. Patients also must have recovered from all treatment-associated toxicities.

Enrolled patients were required to have all baseline disease measuring tests (serum protein electrophoresis, urine protein electrophoresis, and bone marrow biopsy) performed within 28 days of registration in the trial. In addition, all patients were required to be at least 18 years old at enrollment; have a Zubrod Performance Status of 0–2; have received no prior sorafenib; have no significant neurotoxicity at baseline; have no evidence of POEMS; have no active infection requiring antibiotics; have bilirubin ≤1.5 times upper limit of normal (ULN) and/or AST ≤5 times ULN and serum creatinine ≤ULN within 28 days prior to registration; have an ANC >750/*μ*L and platelet count >75,000/*μ*L within 28 days prior to registration; have the ability to take oral medication without crushing, dissolving, or chewing tablets; not be taking cytochrome P450 enzyme-inducing antiepileptic drugs (phenytoin, carbamazepine, and phenobarbital), rifampin, or St. John's Wort. The detailed protocol can be accessed from http://www.mtcancer.org/Protocols.S0434.pdf. Local institutional review boards approved this study. All patients reviewed and signed informed consent forms prior to trial enrollment.

### Study drug treatment

Patients were instructed to swallow two 200 mg tablets of sorafenib with approximately 250 mL (8 oz.) of water each morning and evening (i.e., q 12 h) with or without food, for a daily dose of 800 mg. There were no dose escalations during the study. If a dose reduction was mandated due to toxicity, the dose was not re-escalated, even if toxicity resolved. Dose level reductions were as follows: *level 1* was 400 mg twice daily; *level 2* was 400 mg daily, and *level 3* was 400 mg every other day. Dose modifications were made for clinically significant (grade 3 or 4) hematologic and other toxicities related to protocol therapy. Patients who experienced several toxicities calling for different recommendations were placed on the lowest recommended dose. Any patient requiring further dose modification was removed from the study. Patients continued treatment unless they met any of the following criteria for removal: progression of disease; unacceptable toxicity; delay of treatment for more than 4 weeks for any reason; significant intercurrent illness; or patient's desire to discontinue treatment. A cycle of therapy was defined as 28 days regardless of dose delays.

### Response assessment and criteria

Standard response definitions are used based on the International Uniform Response Criteria for MM 12.

Measurable disease is defined by the presence of quantifiable protein criteria. Acceptable protein criteria are serum M protein ≥1 g/dL (≥10 g/L), quantified by using densitometry on serum protein electrophoresis (SPEP), and/or urine M protein (Bence–Jones Protein) ≥200 mg/24 h (≥0.2 g/24 h), quantified by 24-h urine protein electrophoresis (UPEP). Patients who have both serum M protein levels <1 g/dL and urine M protein levels <200 mg/24 h at baseline may be followed by serum-free light chain (FLC) assay if involved FLC levels ≥10 mg/dL (≥100 mg/L). Oligosecretory and nonsecretory disease patients that do not meet the criteria for measurable disease above may only be assessed for the following objective statuses: stringent complete response, stable disease, and progression.

The objective of the study was to assess the response of the patient according to standardized criteria. Response or progression must be confirmed by a second disease assessment prior to the institution of any new therapy. The second disease assessment may be done at any time. Skeletal survey was not required for assessment of response unless clinically indicated.

### Subject evaluation

Patient evaluations included patient histories/physical examinations, blood and urine tests, bone marrow aspirate and biopsy, imaging scans, weight and performance status, and toxicity. All baseline evaluations were performed prior to beginning study therapy. Patients were required to have their blood pressure monitored weekly until stable or weekly for a minimum of the first 4 weeks of protocol treatment. Weight and performance status was assessed every 4 weeks for the first two cycles. Patients were monitored for toxicity weekly during the first cycle of treatment, then prior to each cycle or at more frequent intervals at the investigator's discretion. Blood and urine tests were performed every 4 weeks for the first two cycles, with complete blood count (CBC) and Prothrobin Time/International Normalized Ratio also being monitored every week for the first cycle. Scans for disease assessment were performed after the first two cycles. After the first two cycles, all disease assessments were monitored every 8 weeks until progression.

### Statistical analysis

It was assumed that sorafenib would not be of further interest if the true response probability (confirmed CR, unconfirmed CR, and PR) was less than 5%. It was also assumed that a true response probability of 20% or more would be of interest in the treatment of patients with relapsed or resistant myeloma.

There was no formal data and safety monitoring committee, as this was a phase II study. Toxicity and accrual monitoring was performed routinely by the Study Coordinator, the Study Statistician, and the Disease Committee Chair. The Study Statistician and the Study Coordinator performed response monitoring. Accrual reports were generated weekly and formal toxicity reports were generated every 6 months. In addition, the Statistical Center, the Adverse Event Coordinator at the Operations Office, and the Executive Officer monitored toxicities on an ongoing basis.

The plan was to initially accrue 20 eligible patients over 10 months with an expected accrual rate of two patients per month. If none of the first 20 patients responded to treatment, the study would be closed and the agent concluded to be inactive. If at least one response was observed, 20 additional eligible patients would be accrued. Five or more responses of the 40 would be considered evidence warranting further study of sorafenib provided other factors, such as toxicity and survival, also appeared favorable. This design had a significance level (probability of falsely declaring an agent with a 5% response probability to warrant further study) of 4.7%, and a power (probability of correctly declaring an agent with a 20% response probability to warrant further study) of 92%.

## Results

### Patient characteristics

**Figure 1:**
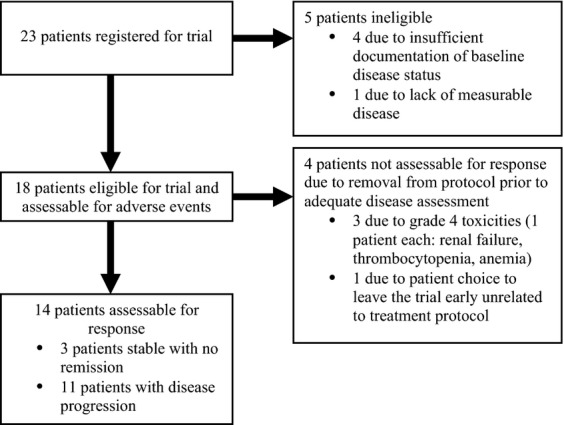
Patient disposition and outcomes.

Twenty-three patients were enrolled between March 2006 and August 2008. Five patients were determined to be ineligible, four of them due to insufficient documentation of baseline disease status and one due to not having measurable disease. Another four patients were not assessable for response due to removal from protocol treatment prior to adequate disease assessment (Fig.1). Median age was 55 years. Only half of the patients were tested for cytogenetics and one-third of the patients tested had apparent abnormalities. Of 17 patients analyzed (one patient amongst the 17 had no serum beta 2 microglobulin levels measured), 8 (47%) were ISS stage I, 7 (41%) stage II, and 2 (12%) stage III (Table 1). All eighteen eligible patients were assessed for toxicity.

**Table 1:** Baseline characteristics of patients

Characteristic	*N* (%)
Sex	
Male	13 (72)
Female	5 (28)
Race	
Caucasian, non-Hispanic	16 (89)
African American	1 (6)
Asian	1 (6)
ISS staging	
Stage I	8 (44)
Stage II	7 (39)
Stage III	2 (11)
Unstaged^1^	1 (6)
Cytogenetics	
Apparent abnormalities^2^	3 (17)
Total patients tested	9 (50)

	Median (range)
Age (years)	54.8 (36.4–70.3)
Prior chemotherapy regimens	4 (2–10)

1 Staging not completed due to lack of serum *β*2 microglobulin.

2 Deletion 17p, deletion 13, hypodiploid 5, and deletion 6 (q13q23).

Of the 18 patients who were assessed for toxicities, 5 (27.8%) received at least one fully dosed cycle, 2 (11.1%) of whom received fully dosed cycles for all cycles administered. An average of 1.5 treatment cycles were received by the group of 18 patients assessed for toxicities.

All eligible patients had a confirmed diagnosis of active MM with measurable protein criteria, which was resistant to or had relapsed from previous treatment, and had received at least two chemotherapy regimens previously. The maximum number of prior regimens administered was 10 with a median of 4 previous regimens. Thus, all patients were heavily pretreated.

Median time from pathological diagnosis to registration was 54.1 months (range 18.9–266.5 months). Cytogenetic studies were performed on nine patients. Of these, three patients had apparent abnormalities (deletion 17p, deletion 13, hypodiploid 5 and deletion 6 [q13q23]). At the end of the trial, three (17%) patients of the 18 assessed were stable with no remission, eleven (61%) experienced increased disease, and four (22%) had inadequate response assessment. Patients were followed up for a maximum of 3 years.

### Safety

The dose of sorafenib administered was 400 mg twice daily for a cycle of 28 days. Patients who experienced toxicities of grade 3 or greater had the drug withheld until the toxicities decreased ≤ grade 1. At least twice a week, toxicities were reassessed. When treatment was reinitiated, the patient was reduced to 400 mg once daily. If a second grade 3 or greater adverse event occurred, the drug was withheld in the same manner and then reinitiated at 400 mg every other day. Treatment was delayed or reduced for hematological toxicities of grade 3 or higher and withheld for nonhematological toxicities of grade 3 or higher until resolution to grade 1. A CBC was obtained weekly until there was a return to no greater than a 25% reduction from baseline values and no sign of infection. All patients have discontinued the protocol treatment as of August 2008.

Eighteen of the 23 patients enrolled in the study were evaluable for adverse events. Toxicities were graded according to CTCAE version 3.0 13. The most common grade 1 toxicity was fatigue, whereas anemia was the most commonly reported toxicity overall. Three grade 4 toxicities were reported including one incidence each of the following: anemia, thrombocytopenia, and renal failure. Grade 3 toxicities included three cases of thrombocytopenia, two cases of leukocytosis and one case each of the following: elevated AST levels, diarrhea, dyspnea, GI distress, hand–foot syndrome, anemia, hyponatremia, hypophosphatemia, chest-wall pain, lower extremity muscle weakness, and rash. Overall, more patients experienced a maximum adverse event of grades 3–4 toxicity than grades 1–2 (11 vs. 6 patients). In total, 60% of patients experienced a grade 3–4 toxicity (Table 2).

**Table 2:** Number of patients with a given type and grade of adverse event per CTCAE v3.0

Adverse event	Grade 1		Grade 2		Grade 3		Grade 4	
			
	*N*	%	*N*	%	*N*	%	*N*	%
Constitutional								
Fatigue	6	33	2	11	0	0	0	0
Fever	2	11	0	0	0	0	0	0
Sweating	1	6	0	0	0	0	0	0
Weight loss	1	6	0	0	0	0	0	0
Hematological								
Hemoglobin	2	11	5	28	1	6	**1**	**6**
Leukocytes	3	17	3	17	2	11	0	0
Neutrophils	1	6	0	0	0	0	0	0
Platelets	2	11	1	6	3	17	**1**	**6**
Renal/genitourinary								
Renal failure	0	0	0	0	0	0	**1**	**6**
Gastrointestinal								
Anorexia	2	11	0	0	0	0	0	0
Constipation	1	6	0	0	0	0	0	0
Diarrhea	1	6	0	0	1	6	0	0
Flatulence	1	6	0	0	0	0	0	0
GI—other	0	0	0	0	1	6	0	0
Mucositis, function: oral cavity	2	11	0	0	0	0	0	0
Nausea	2	11	0	0	0	0	0	0
Taste alteration	1	6	1	6	0	0	0	0
Metabolic/laboratory								
ALT	1	6	0	0	0	0	0	0
AST	2	11	0	0	1	6	0	0
Alkaline phosphatase	2	11	1	6	0	0	0	0
Creatinine	2	11	0	0	0	0	0	0
Hypoalbuminemia	1	6	0	0	0	0	0	0
Hypocalcemia	4	22	0	0	0	0	0	0
Hyponatremia	1	6	0	0	1	6	0	0
Hypophosphatemia	0	0	0	0	1	6	0	0
Pulmonary/upper respiratory tract								
Cough	2	11	0	0	0	0	0	0
Dyspnea	1	6	1	6	1	6	0	0
Voice changes	1	6	0	0	0	0	0	0
Syndromes								
Flu-like syndrome	0	0	1	6	0	0	0	0
Infection								
Lung infection, 3–4 ANC: upper airway	0	0	1	6	0	0	0	0
Dermatologic								
Acne	1	6	0	0	0	0	0	0
Alopecia	2	11	1	6	0	0	0	0
Dermatology—other	1	6	0	0	0	0	0	0
Dry skin	1	6	0	0	0	0	0	0
Hand–foot	1	6	3	17	1	6	0	0
Pruritus	2	11	0	0	0	0	0	0
Rash	3	17	0	0	1	6	0	0
Musculoskeletal								
Muscle weakness: lower extremities	0	0	0	0	1	6	0	0
Auditory/ear								
Tinnitus	0	0	1	6	0	0	0	0
Hemorrhage/bleeding								
Hemorrhage—other	1	6	0	0	0	0	0	0
Lung hemorrhage: nose	1	6	0	0	0	0	0	0
Pain								
Lung pain: chest wall	0	0	0	0	1	6	0	0
Musculo. pain: back	0	0	1	6	0	0	0	0
Musculo. pain: muscle	1	6	1	6	0	0	0	0
Musculo. pain: bone	1	6	0	0	0	0	0	0
Neuro pain: head/headache	2	11	0	0	0	0	0	0
Pain other	0	0	1	6	0	0	0	0
Neurological								
Confusion	1	6	0	0	0	0	0	0
Dizziness	1	6	0	0	0	0	0	0
Mood alteration: depression	2	11	0	0	0	0	0	0
Neuropathy-motor	1	6	0	0	0	0	0	0
Neuropathy-sensory	0	0	1	6	0	0	0	0
Psychosis	0	0	1	6	0	0	0	0
Cardiac arrhythmia								
Palpitations	1	6	0	0	0	0	0	0

Grade 4 toxicities are bolded.

### Efficacy

The study opened for accrual in March 2006. It was temporarily closed to accrual in July of the same year, after meeting its first stage accrual goal and was then permanently closed in August 2008.

**Figure 2:**
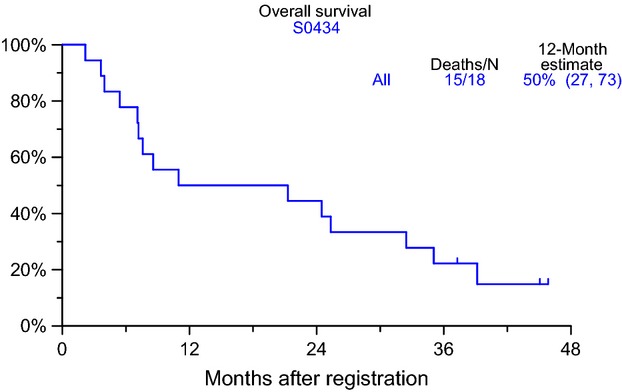
Overall survival among eligible patients (data as of 6 February 2013).

**Figure 3:**
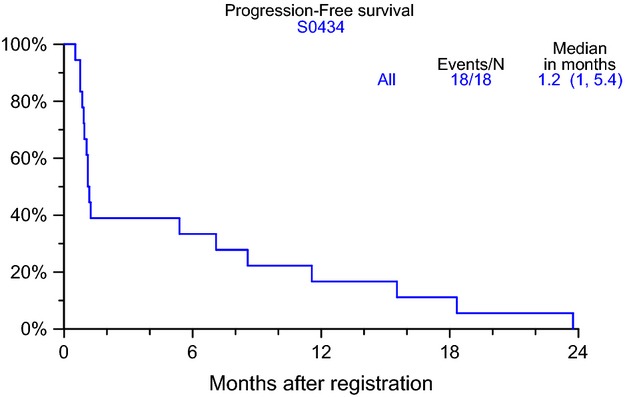
Progression-free survival among eligible patients (data as of 6 February 2013).

No partial or complete response was observed on this study among the 14 patients who were assessable for responses. Four patients were not assessable for response due to removal from protocol treatment prior to an adequate disease assessment. All 18 patients progressed, 11 of whom discontinued treatment due to progression. The remaining seven patients went off study for the following reasons: adverse events (4), patient refusal (1) and other, not protocol specific reasons (2). None of the patients died while they were on treatment. Overall survival (Fig.2) at 12 months was 50% (95% CI 27–73) and median progression-free survival (PFS) was 1.2 months (95% CI 1.0–5.4). PFS rates were ∼33% at 6 months and 17% at 1 year (Fig.3).

## Discussion

Sorafenib is a targeted therapeutic drug that is a member of the tyrosine kinase inhibitor family. Recently its role is also demonstrated in downregulating antiapoptotic MCL-1 protein 14–16. It also blocks angiogenesis via the Ret/Ras pathway.

Various phase II and III trials using sorafenib as a single agent or in combination therapy have been conducted in breast cancer, non–small-cell lung cancer, metastatic melanoma, sarcoma, thyroid cancer, and head and neck cancer 2. As a single agent it has been used in the treatment of renal cell carcinoma and hepatocellular carcinoma 17–19. Sorafenib is approved as a first-line treatment option for relapsed or medically unresectable stage IV predominately clear cell renal carcinoma 20. Phase I and II trials report sorafenib, when used as a part of a multidrug regimen, showed potential to produce partial response (PR) to treatment of ovarian and breast cancer 21. More recently, it was shown to be comparable to other single antiangiogenic drugs in the treatment of metastatic breast cancer, and has in vitro activity against several malignant pleural mesothelioma lines 2,22. Sorafenib is relatively well tolerated and has been shown to have synergistic activity with dexamethasone, bortezomib, and rapamycin in the in vitro killing of myeloma cells lines 5.

In our study, sorafenib was investigated as a single agent in patients with refractory or relapsed (RR) MM as SWOG protocol S0434. The results showed that, of the 14 patients assessable for response, 3 were stable and the remaining 11 showed progression of disease. Treatment was well tolerated by most patients. This is in agreement with the recently published dual center open label prospective study with symptomatic relapsed or refractory MM 23. Dose administered was the same except that the regimen was for 13 cycles as opposed to ours which was open ended until toxicities or progression or patient choice to end the treatment. The toxicity profiles in both studies are largely similar indicating that sorafenib is generally well tolerated.

In this study, only three patients reported grade 4 toxicities (thrombocytopenia, anemia, and renal failure). There were no deaths during the duration of this study.

In the phase I study using larger doses of sorafenib, 600 and 800 mg twice daily, skin rash, hand–foot syndrome, diarrhea, nausea, hypertension, and fatigue were observed as the most common adverse events. Grade 3- and 4-related toxicities were observed in 60% of the patients and included amylase and lipase elevation without association with symptoms of pancreatitis and hand–foot syndrome. Myelosuppression was not a common adverse event of sorafenib and therefore the use of granulocyte colony stimulating factor if necessary was acceptable.

The trial did show that no partial or complete response was observed of the 14 patients who were assessed for responses. At least three patients remained with stable disease. Overall survival at 12 months was 50% (95% CI 27–73) and median PFS was 1.2 months (95% CI 1.0–5.4).

The trial was limited by a small number of heavily pretreated patients diagnosed with RR MM. The lack of response has also been observed in recent metastatic breast cancer trials using sorafenib as a single agent 2,24.

There are several open trials with sorafenib in combination with current MM therapies. The Mayo Clinic trial using sorafenib, lenalidomide, and dexamethasone for patients with relapsed or refractory MM was closed to accrual prior to opening the phase two portion due to study design and toxicity 25. The two open trials are no longer recruiting for RR MM. Both are phase I/II, one with the combination of sorafenib/bortezomib (completed accrual) from Sarah Cannon Research Institute 26. The second trial by Kumar et al. used sorafenib/everolimus to determine the maximum tolerated doses (MTD) of the two drugs in combination and the efficacy of the combination 27. This trial accrued 26 patients with lymphoma or MM. The MTD from this trial was determined to be 200-mg sorafenib daily. Thirteen patients had grade 3 or 4 hematologic toxicities. This trial did demonstrate activity and results are interesting. However, results from this trial cannot be compared to ours because this trial had only 2 patients with MM and 24 patients had lymphoma.

Treatment of MM with patient-specific and novel therapies can potentially make MM a chronic disease and ultimately provide a cure 28,29. Current therapies for MM intend to improve patient survival and quality of life with minimal toxicity 30.

This study was conducted when very few novel agents were available for testing as single agent therapy. The intention of this trial was to identify the activity of anti-Raf agent sorafenib in heavily pretreated RR MM patients and determine its safety profile for future combination drug trials.

In our study it seems that sorafenib has very limited activity as a single agent in unselected patients with RR MM. Nonetheless, the observed well-tolerated side effects profile in our study and most importantly the ease of administration as an oral agent makes sorafenib interesting candidate to investigate further in combination therapy with current or future standard antimyeloma treatments especially as a late-line option. Moreover, it may be possible that therapeutic response may be observed if patients are screened for the RAS/BRAF/VEGFR mutations. These mutations have been observed to be present both in newly diagnosed and RR MM 8,9. Andrulius et al. correlated the BRAF mutation status in primary tumor samples from 379 myeloma patients with disease outcome. They found that the mutation carriers when compared with controls had a significantly higher incidence of extramedullary disease and a shorter overall survival. In this study, one patient with confirmed BRAF V600E mutation and relapsed myeloma with extensive extramedullary disease who was refractory to all approved therapeutic options rapidly and durably responded to low doses of the mutation-specific BRAF inhibitor vermurafenib 31. To better address the efficacy of this class of drugs, large-scale investigation of BRAF inhibitors in *BRAF*-mutant tumors is under way. The Basket study, open-label, phase II study of vemurafenib in patients with *BRAF* V600 mutation-positive cancers, including myeloma (NCT01524978) is ongoing 32.

Ras mutations have been noted in 35% to 50% of MM patients. Mutations of K-Ras has been associated with poor survival 33. The correlation of these mutations with therapeutic results needs to be further studied.

Selection of patients based on genetic mutations will enrich for a population that could benefit and respond from sorafenib or other Raf or Ras inhibitors. This will provide a novel avenue of patient-specific personalized therapy.
